# Idiopathic Multiple Localized Lipoatrophy Mimicking Amyotrophic Lateral Sclerosis

**DOI:** 10.7759/cureus.74887

**Published:** 2024-12-01

**Authors:** Shuhei Mashimo, Ayano Matsuoka, Keiji Tanese, Osamu Kano, Akira Ishiko

**Affiliations:** 1 Department of Dermatology, Toho University School of Medicine, Tokyo, JPN; 2 Department of Neurology, Toho University School of Medicine, Tokyo, JPN

**Keywords:** amyotrophic lateral sclerosis, humoral immunity, idiopathic localized lipoatrophy, inflammatory-type, plasma cell

## Abstract

Localized lipoatrophy is a rare condition characterized by the localized loss of subcutaneous adipose tissue. It may occur idiopathically without specific triggers. The pathogenesis of idiopathic localized lipoatrophy remains largely unknown. We present the case of a 53-year-old Japanese woman with multiple localized lipoatrophy who exhibited upper motor neuron signs clinically and panniculitis histologically. She was initially suspected to have amyotrophic lateral sclerosis due to progressive left limb volume loss. Histologically, the lesions showed adipocyte destruction accompanied by predominant plasma infiltration.

## Introduction

Lipoatrophy syndromes are a heterogeneous group of disorders characterized by the lack of subcutaneous fat tissue [1]. These syndromes are classified based on the extent and location of subcutaneous fat loss as follows: generalized type, where lesions affect almost the entire body; partial type, where lesions are confined to specific anatomical sites; and localized type, where lesions are restricted to a limited area [2]. Here, we report a case of multiple localized lipoatrophy, which demonstrates upper and lower motor neuron signs that may indicate amyotrophic lateral sclerosis (ALS) in neurological findings and predominant infiltration of plasma cells in adipose tissue in histological findings.

## Case presentation

A 53-year-old Japanese female presented with a chief complaint of skin depressions on the left upper extremity and lower extremity. She first noticed the lesion on the left upper arm about two years ago, followed by the gradual appearance of multiple lesions on the left shoulder, left lower abdomen, and left lower extremity. There was no history of injections or trauma to the skin lesions. In addition, there was no history of viral infection or vaccination that could have triggered the lesions, nor was there any occupational history suggestive of potential causes for neurological abnormalities. Her medical history included a previous surgical resection for left breast cancer, and she is undergoing hormone therapy with tamoxifen. She has no history of hyperlipidemia or diabetes. The familial history of the disease was absent.

**Figure 1:**
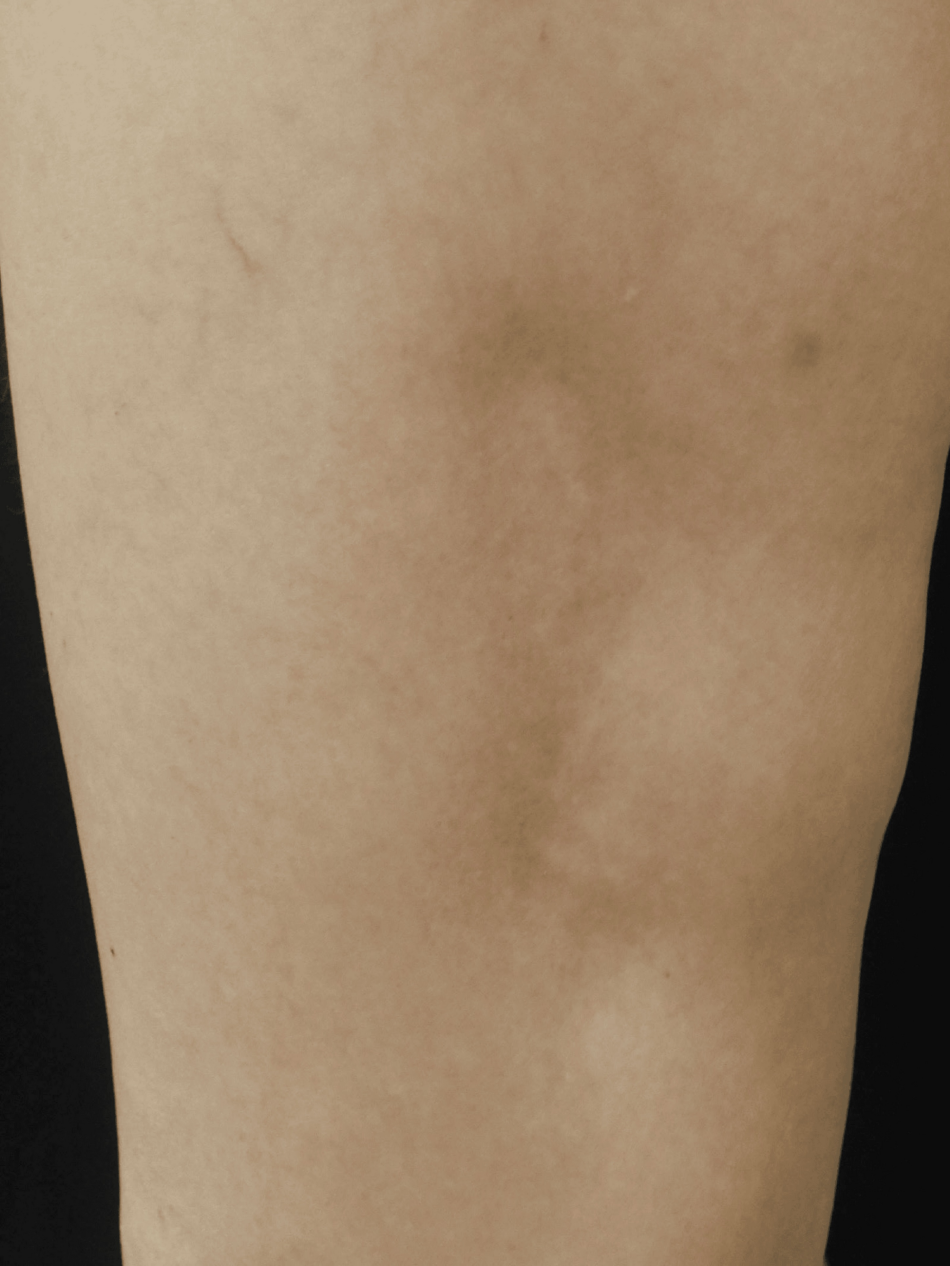
Skin lesion on the left thigh The figure shows depressions on the left thigh.

**Figure 2:**
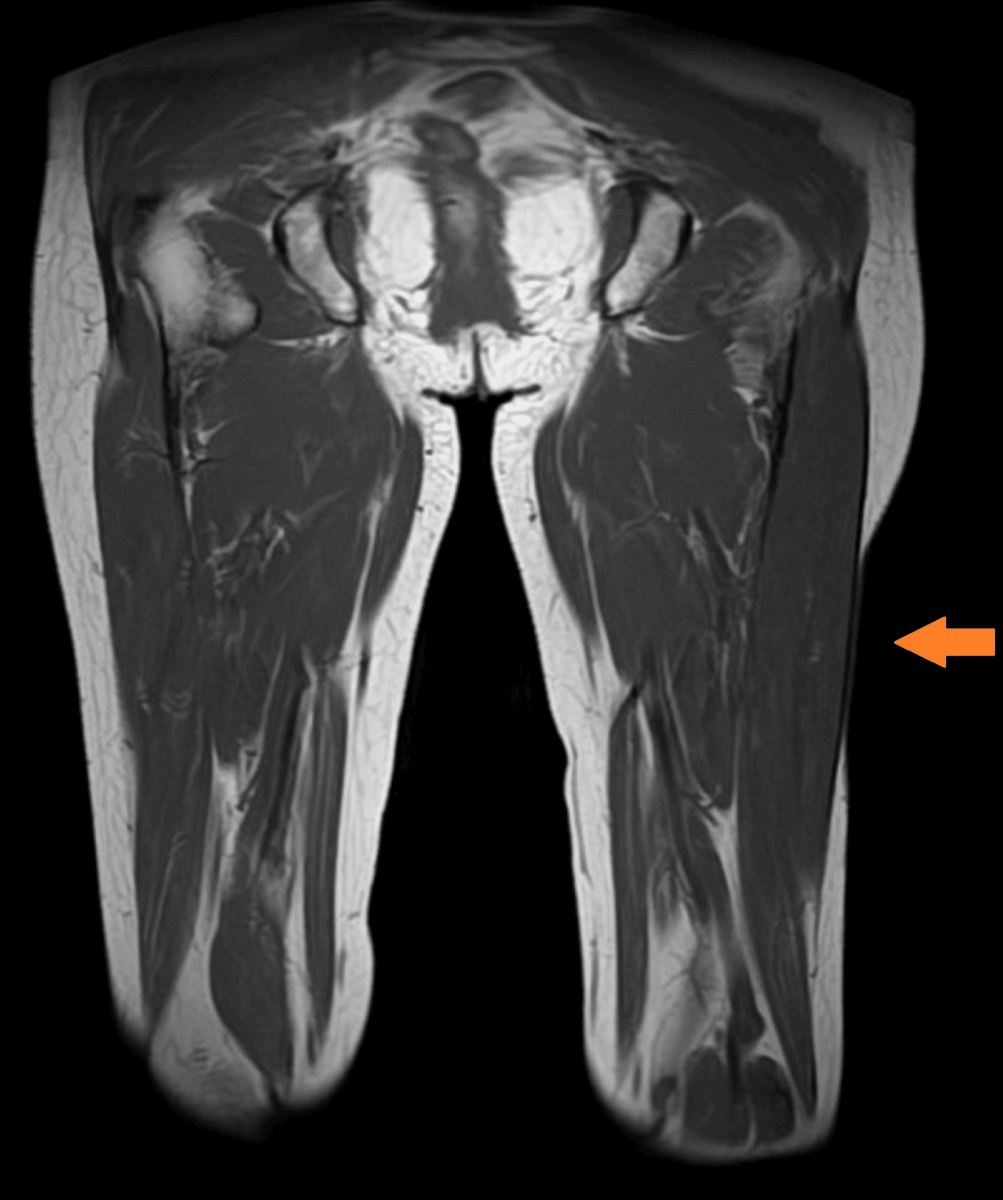
T1-weighted images on MRI of the thigh The figure shows skin depression on the left thigh. No muscle atrophy was observed, but there was a noted loss of approximately 8 cm of subcutaneous adipose.

On clinical examination, the skin exhibited a diffused indentation on the left upper arm, and multiple skin depressions with a maximum diameter of 8 cm were observed on the left thigh (Figure 1), left lower extremity, left shoulder, and left lower abdomen. Furthermore, tendon reflex was increased, and Hoffmann’s/Trömner’s reflex was positive in the upper limb. There were no clinical findings suggesting dystonia. Blood tests showed no abnormalities in the complete blood count or biochemical tests, including immunoglobulin. Anti-nuclear and anti-double-stranded DNA antibodies were negative. Nerve conduction and electromyography showed no abnormal electrophysiological findings. Magnetic resonance imaging (MRI) of the head and cervical spine were normal. T1-weighted MRI of the limb revealed no evidence of muscle atrophy, although fat atrophy was observed in the lesion (Figure 2).

**Figure 3:**
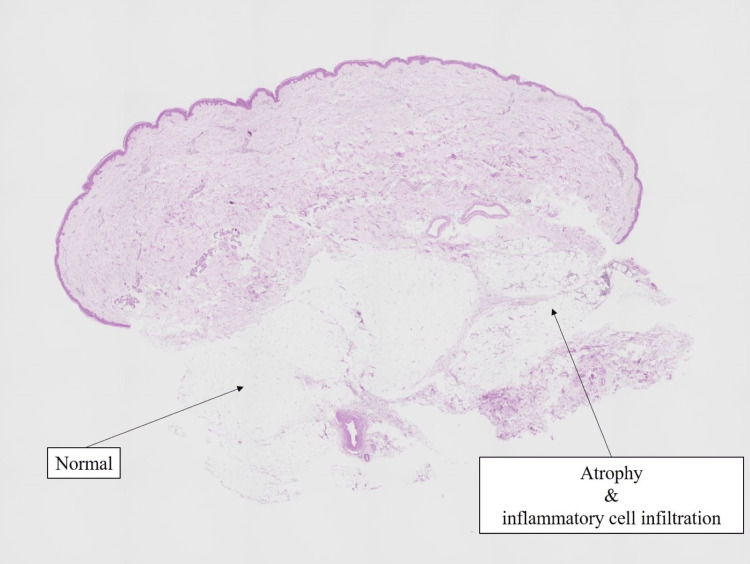
Low-power view of the skin biopsy Hematoxylin and eosin (H&E) staining (×4). The adipose tissue of the lesion showed atrophy and inflammatory cell infiltration compared to normal tissue (right side of the specimen).

**Figure 4:**
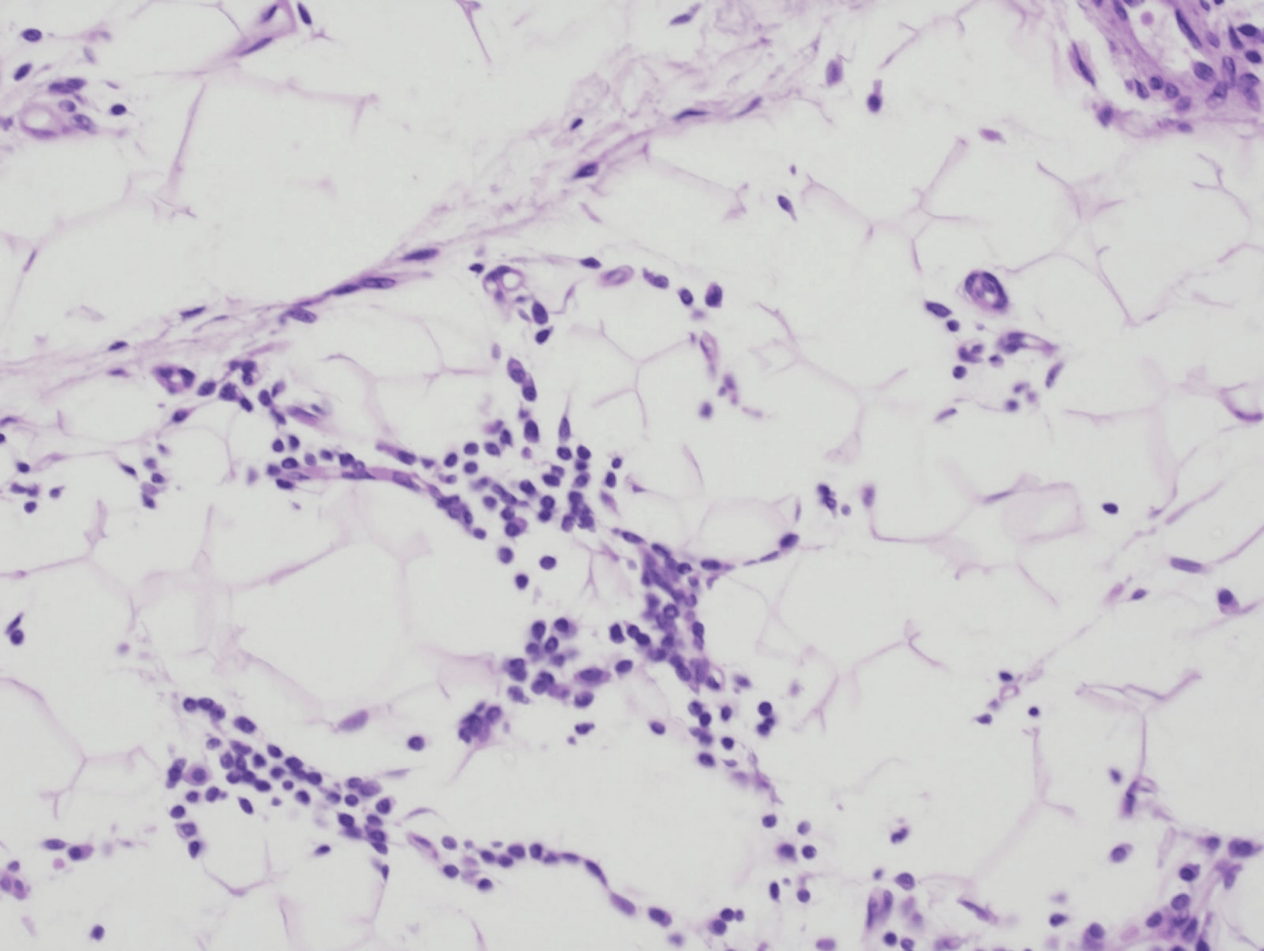
High-power view of the skin biopsy Hematoxylin and eosin (H&E) staining (×200). Mononuclear inflammatory cells infiltrated adipose tissue.

**Figure 5:**
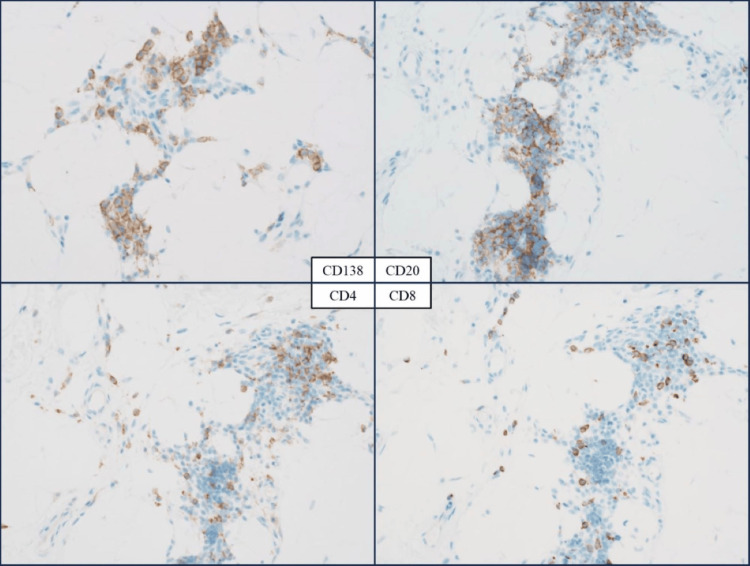
High-power view of the immunological pathology CD138, CD20, CD4, and CD8 staining (×200). The inflammatory cells infiltrating the adipocytes were predominantly CD138+ plasma cells and CD20+ B cells, with relatively few CD4+ and CD8+ T cells.

Since there were no further findings strongly suggestive of ALS, a skin biopsy was performed at the border between the depressed area and the healthy area. Histological examination revealed that the lesional adipose tissue was atrophic compared to normal tissue (Figure 3). At high-power magnification, adipocyte degeneration and inflammatory cells, including lymphocytes and plasma cells, within the adipose tissue were observed (Figure 4). Immunohistochemical analysis revealed the presence of CD138+, CD20+, CD4+, and CD8+ cells, with a notable prevalence of CD138+ cells (Figure 5). Finally, a diagnosis of idiopathic localized lipoatrophy was made based on the clinical presentation of focal and asymmetric idiopathic lipoatrophy, coupled with the pathological findings of adipose tissue atrophy and adipocyte degeneration. The history of tamoxifen administration was also ruled out as a causative factor, as there were no concurrent metabolic disorders associated with lipolysis, and the symptoms were observed only unilaterally. The influence of tamoxifen, which the patient had been taking, cannot be completely excluded as a causal factor since it has been suggested to enhance the polarization of CD4+ T cells toward the Th2 phenotype [3]. However, since there are no reports of localized lipoatrophy occurring under the same condition and the distribution of the lesions in this case is asymmetric as a drug-induced skin lesion, the causal relationship is unlikely.

## Discussion

Localized lipoatrophy is a rare condition characterized by the loss of subcutaneous adipose tissue in the limited areas of the body [2]. It may occur because of adverse drug reactions, physical compressions such as injection or injury, or conditions that cause panniculitis. However, it may also occur idiopathically without specific triggers [2]. A comprehensive understanding of the pathobiological mechanisms of idiopathic lipoatrophy remains elusive. In this case, histopathological examination of the lipoatrophy lesion demonstrated inflammatory cells within adipose tissue, accompanied by adipocyte degeneration. Immunohistochemical staining further revealed that CD138-positive plasma cells represented a predominant cellular component.

Localized lipoatrophy has been histologically classified into two subtypes. In a study involving 11 patients, Peters and Winkelmann. identified these as the involutional and inflammatory types [4]. The involutional type is characterized by fat lobules composed of small to medium-sized lipocytes set against a background of hyaline material, with a scarcity or absence of inflammatory cells. In contrast, the inflammatory type is characterized by scattered focal inflammatory cell infiltration within the normal-appearing adipose tissue. Clinically, all involutional-type cases exhibited solitary lesions, whereas all inflammatory-type cases exhibited multiple lesions [4]. Our case is consistent with an inflammatory-type lipoatrophy both clinically and histopathologically.

As lymphocytes, histiocytes, and plasma cells have been reported to infiltrate the subcutaneous tissue [4], it has been postulated that these immune cells may be involved in the pathogenesis of inflammatory localized lipoatrophy. However, the precise type of cells predominantly infiltrating the tissue has yet to be studied. The results of immunostaining in the histopathology specimen of this case indicate that plasma cell-mediated humoral immunity plays an important role in the pathogenesis of inflammatory-type idiopathic lipoatrophy. Supportively, a significant proportion of reported patients presenting with inflammatory-type localized lipoatrophy have been found to exhibit serum antinuclear or anti-double-stranded DNA antibodies, which may clinically suggest an association with autoimmune diseases [2]. These findings indicate that autoimmune mechanisms may play a more prominent role in the pathogenesis of multiple forms of localized lipoatrophy. In this case, although both antinuclear and anti-dsDNA antibodies were negative, the histopathological evidence of plasma cell and B cell infiltration in a localized area of adipose tissue might have contributed to a focal autoimmune reaction associated with humoral immunity.

Neurologically, lipoatrophy may complicate peripheral neuropathy caused by laminopathies themselves or by diabetes [5]. However, reported cases of inflammatory-type localized lipoatrophy with concomitant specific organ symptoms are limited. Moreover, there have been no reports of cases presenting with upper motor neuron signs, as observed in this case. Previous studies have suggested that endoplasmic reticulum stress and cerebral phospholipid abnormalities may be involved in laminopathy [6], which could potentially lead to upper motor neuron disorders. Furthermore, lipoatrophy is rarely associated with muscle atrophy [7], although this was ruled out in the present case based on normal electromyography and MRI findings. While further accumulation of additional studies is necessary, the features demonstrated in this case and these reports suggest that lipoatrophy may affect the surrounding tissues.

## Conclusions

This is the first case of inflammatory-type lipoatrophy with neurological impairment without subjective neurological symptoms. As the pathophysiology of lipoatrophy is unknown, we may have to pay more attention to the neurological findings of this disease.
